# The iceman cometh

**DOI:** 10.1186/2041-2223-3-8

**Published:** 2012-04-16

**Authors:** Mark A Jobling

**Affiliations:** 1 Department of Genetics, University of Leicester, University Road, Leicester LE1 7RH, UK

## 

Invited to give a seminar in Lausanne, I did some research about the place. According to Wikipedia (so it must be true), it is the smallest city in the world to possess a metro system. And very useful this proved to be, the day after my talk, carrying me with driverless Swiss efficiency from the edge of Lake Geneva up the steep hill to the Saturday market, where I bought excellent bread and a *saucisse de cerf* (salami made from deer meat) to take home.

Lausanne is a fine city, and seems like a good place to live (“like France - only cleaner,” said my host). It perches above the beautiful water opposite Evian-les-Bains, spectator to a dramatic Alpine panorama, and with the wonderful Jura mountains on its western shoulder. Its charm has attracted a romantic collection of celebrities – recent denizens include Coco Chanel, Georges Simenon and Charlie Chaplin. Voltaire gave his name to a street, Lord Byron stayed at the Hôtel d’ Angleterre (almost drowning in the Lake), and Charles Dickens wrote *Dombey and Son* while living at the Villa Rosemont.

We know a lot about Dickens’ stay from his frequent letters, recorded in his friend John Forster’s biography [1]. September 1846 found him on an outing to the Alps, ascending via the nineteenth century equivalent of the metro (carriages and mules) to the Great St Bernard pass. They stayed at the hospice, “supping, thirty strong, in a rambling room with a great wood-fire… and a grim monk, in a high black sugar-loaf hat with a great knob at the top of it, carving the dishes”.

However, Dickens’ letter reminds us that life in the Alps is not all ease and elegance, as he makes a grim discovery: “Beside the convent, in a little outhouse with a grated iron door which you may unbolt for yourself, are the bodies of people found in the snow who have never been claimed and are withering away – not laid down, or stretched out, but standing up, in corners and against walls; some erect and horribly human, with distinct expressions on the faces … holding ghastly possession of the mountain where they died”.

These arresting images bring to mind a more recent find, a few hundred kilometres to the east. If Ötzi the Iceman had been discovered in the mid-nineteenth century, he too might well have ended up decaying in a little outhouse. The two German tourists who encountered him melting out of the ice in the Ötztal Alps on the Austrian-Italian border in September 1991 [2] assumed his was a recent death. When they reached their alpine lodge at the end of the day, they asked if any local people were missing. But it turned out that although the corpse was indeed local, it was certainly not recent: carbon dating of grass samples gave a late Neolithic age of 5300 years. Data from sequence analysis of the Iceman’s genome have now revealed new information about his ancestry [3].

Ötzi rapidly became a celebrity, attracting an appropriate amount of scandal and bizarre headlines – one woman insisted that the body was that of her long-lost father, another claimed that she was his reincarnation, while a third wrote to request that she be impregnated with the Iceman’s sperm. A report that Ötzi’s genitalia were apparently missing fuelled the idea that he was in fact an Egyptian mummy (often castrated); this formed part of the evidence in a book claiming that the discovery was an elaborate and lucrative archaeological fake. The existence of the understandably shrivelled organs was confirmed in a subsequent physical examination. Informal and unjustified use of the species name *Homo tirolensis* provoked strong academic criticism [4], and the project director Konrad Spindler’s embroidered account of how Ötzi met his death led Spindler to be branded a ‘frustrated novelist’.

The first genetic analyses were challenging, because despite the remarkable degree of preservation of the body, its DNA was severely degraded. PCR-based analysis of hypervariable segment I of mitochondrial DNA (mtDNA) [5] yielded a convincing result after much trouble: two nucleotide transitions were observed with respect to the Cambridge reference sequence, indicating that the mtDNA belonged to haplogroup K, present in Europe at a frequency of a few percent. One of the study’s authors, Bryan Sykes, unveiled a living person sharing the same sequence, a Marie Moseley of Bournemouth, originally from Ireland, who claimed Ötzi as a travelling Irishman who had gone astray on his European travels.

It was the advent of next-generation sequencing that led to higher-resolution and more interesting results, because these methods are well suited to analysing very short DNA fragments. We now have a complete mtDNA sequence [6], which belongs to a previously unknown branch of haplogroup K1, respectfully dubbed K1ö. Most recently, nuclear DNA sequences covering 96% of the reference genome have been described [3]. Phenotypic predictions for Ötzi include blood group O (perhaps it should be Ö), brown eye colour and lactose intolerance. Identification of the bacterium *Borrelia burgdorferi* from the metagenome indicates that the Iceman probably suffered from the tick-borne disease Lyme borreliosis. His Y chromosome haplogroup, G2a4, has its highest frequencies in Corsica and Sardinia, and principal components analysis of his autosomal genome together with SNP data from modern populations also indicates affinity to modern Sardinians. This might suggest that Ötzi was a Sardinian immigrant to the Alps, but alternatively might reflect deep shared ancestry between past Sardinian and Alpine populations – additional ancient sequences from the regions would help to throw light on this. Stable isotope compositions of teeth and bones can indicate where a person grew up and lived, and in Ötzi’s case these show that he was local to the valleys above which his body was found [7].

DNA methods were also successfully applied to the contents of Ötzi’s intestinal tract [8], revealing that his last meals included bread and dried deer-meat – as I discovered, items that are still available from an alpine market today.

## References

[B1] Forster J The Life of Charles Dickens (originally published 1872–4) 2005 Uitgeverij Diderot

[B2] Fowler B Iceman: Uncovering the Life and Times of a Prehistoric Man found in an Alpine Glacier 2000 Macmillan, London

[B3] Keller A Graefen A Ball M Matzas M Boisguerin V Maixner F Leidinger P Backes C Khairat R Forster M New insights into the Tyrolean Iceman's origin and phenotype as inferred by whole-genome sequencing Nat Commun 2012 3 698 22426219 10.1038/ncomms1701

[B4] Editorial Naming people lightly Nature 1995 373 176 7816115 10.1038/373176b0

[B5] Handt O Richards M Trommsdorf M Kilger C Simanainen J Georgiev O Bauer K Stone A Hedges R Schaffner W Molecular genetic analyses of the Tyrolean Ice Man Science 1994 264 1775 1778 10.1126/science.8209259 8209259

[B6] Ermini L Olivieri C Rizzi E Corti G Bonnal R Soares P Luciani S Marota I De Bellis G Richards MB Complete mitochondrial genome sequence of the Tyrolean Iceman Curr Biol 2008 18 1687 1693 10.1016/j.cub.2008.09.028 18976917

[B7] Muller W Fricke H Halliday AN McCulloch MT Wartho JA Origin and migration of the Alpine Iceman Science 2003 302 862 866 10.1126/science.1089837 14593178

[B8] Rollo F Ubaldi M Ermini L Marota I Otzi's last meals: DNA analysis of the intestinal content of the Neolithic glacier mummy from the Alps Proc Natl Acad Sci U S A 2002 99 12594 12599 10.1073/pnas.192184599 12244211 PMC130505

